# Grey-Blue Regression in Melanoma In Situ—Evaluation on 111 Cases

**DOI:** 10.1155/2011/180980

**Published:** 2011-03-23

**Authors:** S. Bassoli, S. Borsari, C. Ferrari, F. Giusti, G. Pellacani, G. Ponti, S. Seidenari

**Affiliations:** Department of Dermatology, University of Modena and Reggio Emilia, Modena, Italy

## Abstract

As fibrosis and melanosis are often seen in malignant melanoma, the presence of dermoscopic signs of regression may represent a clue for the diagnosis of malignancy. Our aim was to assess the frequency and extent of 11 dermoscopic features of regression evaluating dermoscopic images of 111 melanomas in situ (MIS). Regression structures (grey-blue areas, white areas, peppering, and/or blue-whitish veil) were present in 80.1% of the lesions. Approximately 80% of the lesions showed regression of dermoscopic structures and light brown areas. Most lesions showed the presence of grey-blue areas (74.7%), whereas peppering was observable in 30.6% of MIS. Areas of fibrosis were mainly observable as structureless areas with a pinkish hue (50.4%). Based on our data, the reticular pattern of blue regression and light brown areas can be considered a significant discriminator and a reliable predictor of MIS.

## 1. Introduction

Regression structures are employed for detection of malignant melanoma (MM) by dermoscopy and are defined by the presence of white and grey-blue areas in the dermoscopic lesion image [1–3]. White areas have been described as areas whiter than the surrounding skin (white scar-like areas), whereas grey-blue areas appear as homogeneous or nonhomogeneous areas (blue pepper-like granules or globules) with a bluish color [3–5]. White and blue colours in a melanocytic lesion may be simultaneously or separately present, suggesting a different therapeutic approach; moreover, their distribution and their location in the centre or at the periphery of the lesion may represent an aid to distinguish between different subtypes of melanocytic lesions [3]. Histopathologically, blue regression may correspond to an abundance of melanin pigment, within either melanophages or pigmented melanocytes in the dermis [6], whereas in the presence of the classic blue-whitish veil, histopathology shows an acanthotic epidermis with compact orthokeratosis and focal hypergranulosis above sheets of heavily pigmented melanocytes or melanophages in the dermis [6–8]. Lesions with regression often carry great histopathological controversy. However, the presence of dermoscopic signs of regression may represent a clue for the diagnosis of malignant lesions. In our previous paper, comparing the regression features observed in 85 MIS, 85 invasive MMs, and 85 nevi with equivocal dermoscopic aspects, the presence of a blue-whitish veil was more frequently seen in invasive MMs, while grey-blue areas with a reticular pattern represented a significant descriptor for MIS [9]. The aim of the present study is to expand the study population with respect to our previous publication, evaluating the dermoscopic features of 111 lesions for the presence of 11 parameters of regression. 

## 2. Material and Methods

The study population included 111 consecutive lesions, excised at the Department of Dermatology of the University of Modena in the years 2003–2009, where we assessed the presence or absence of 11 parameters of regression (Table 1). The dermoscopic images were evaluated by 3 experienced dermatologists. Final judgment on each descriptor derived from a consensus of at least 2 of 3 observers.

**Figure 1:**
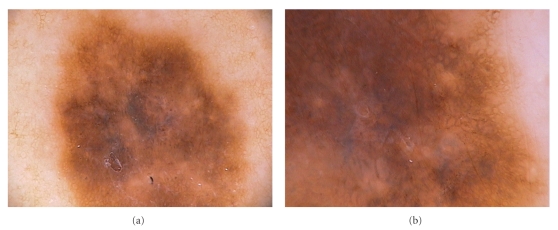
Reticular grey-grey-blue areas. (a) In situ melanoma with reticular grey-blue pigmentation in the centre of the lesion (30-fold magnification). (b) Detail of a 50-fold magnified in situ melanoma, where reticular grey-grey-blue areas are well detectable.

**Figure 2:**
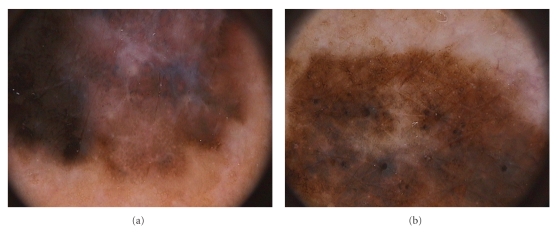
Association of different dermoscopic aspects of regression. (a) In situ melanoma showing white and pink areas as well as blue structureless areas (original magnification ×20). (b) Dermoscopic image of an in situ melanoma showing blue structureless and globular areas associated with peppering (original magnification ×20).

Dermoscopic descriptors. Regression was considered present when grey-blue areas, peppering, blue-whitish veil, and/or white areas were present. Grey-blue areas were further described as being globular, reticular, or structureless (Figure 1). “Globular grey-grey-blue areas” were represented by aggregated grey-blue globules, whereas the term “reticular grey-grey-blue areas” defined a coarse blue-grey net, with thick grey-blue lines and large holes, corresponding to white or pink regression areas (Figure 2). Although often present in lesions with a network, reticular grey-blue pigmentation does not correspond to the pigment network, which has smaller meshes and thinner lines. Pink lesion areas, as areas of the lesion lighter than the surrounding skin with a pinkish shade, were also observed. For blue and white areas, both their presence and their extent were assessed. Unlike in our previous paper, the presence of the blue-whitish veil was only considered when the lesion was clinically palpable. Moreover, we also considered the presence of regression of dermoscopic structures and light brown areas defined as light brown structureless irregular areas especially located at the periphery of the lesions [10] “Regression of dermoscopic structures” was visible when, within a structured area, there was a fading of net, globules, or pigmentation giving rise to light brown structureless areas. 

## 3. Statistics

Absolute and relative frequencies of the observations were obtained for each regression parameter. Absolute and relative frequencies of lesions showing only one or a combination of the evaluated dermoscopic features were calculated on the number of lesions presenting regression. 

## 4. Results

The study included a total of 111 melanocytic lesions diagnosed as MIS according to conventional histopathological criteria. These were located on the trunk and limbs of 110 patients (50 female and 60 male, mean ± SD age 56.4 ± 15.7 years).

Table 2 shows the results of the assessment of regression parameters in 111 MIS. Regression structures (i.e., grey-blue areas, white areas, peppering, and/or blue-whitish veil) were present in 80.1% of the lesions. Approximately 80% of the lesions showed regression of dermoscopic structures and light brown areas. Most MIS showed the presence of grey-blue areas (74.7%), whereas peppering was observable in 30.6% of MIS. Areas of fibrosis seldom appeared as white areas (10.8%) but were rather observable as structureless areas with a pinkish hue (50.4%). In our series of MIS, the blue-whitish veil was noticed in only 1.8% of the cases (Figures 1 and 2).

Table 3 shows data regarding pattern and extent of grey-grey-blue areas. The reticular pattern was the most frequently observed along with the structureless pattern. Grey-grey-blue areas involved 10–50% of the lesion area in more than half of the cases.

Table 4 shows the number of regression parameters in the study population. One regression parameter was present in 64% of MIS. The presence of two regression descriptors was found in 25.8% of MIS, whereas 3 regression parameters or more were observed in 10.1% of cases in our population.

When subdividing our cases according to the diameter of the lesion, we observed a higher percentage of lesions showing regression, in particular grey-grey-blue areas and white/pink structures, in MIS with a diameter >1 cm (Table 5). 

## 5. Discussion

Blue-white structures, corresponding to histological regression phenomena, are one of the most interesting dermoscopic features commonly seen in melanomas and represent a clue for the diagnosis [3, 11–16]. Blue-white structures in dermoscopy include white areas, scar-like or associated with a reddish shade, grey-grey-blue areas, or a combination of both. Histologically, white areas correspond to fibrosis, whereas grey-grey-blue areas correspond to melanophages in the dermis [17, 18]. Dermoscopically, grey-blue areas and the blue-whitish veil are difficult to distinguish, and this causes a low reproducibility in the identification of these features [12–16, 19–23]. Therefore, they are generally considered together and described by the unifying definition of blue structures [24].

 In our previously published paper, comparing the dermoscopic features of regression in equivocal lesions, MIS, and invasive MMs, we found that regression is not only more frequent and extensive in invasive MMs with respect to in situ lesions, but it also has a different morphological expression [9]. 

In the present study, features of regression were re-evaluated on 111 lesions histologically diagnosed as MIS, assessing the extent, the morphology, and the correlation of regression features, also according to the diameter of the lesion. The results substantially confirm the data we obtained in the previous study: regression structures were present in 80% of cases and were mainly represented by grey-blue areas with a reticular pattern and structureless light brown pigmentation. Grey-blue areas can represent the only expression of regression features, or, less frequently, they can be associated to peppering. Unlike in our previous study, the presence of the blue-whitish veil was only considered when the lesion was clinically palpable; therefore, only few lesions with this feature were identified by our evaluation.

We also observed that regression features, as expressed by the presence of grey-blue areas and white/pink structures are more frequently observable in large lesions indicating that the regression process evolves with the radial growth of the MM.

 In MIS, grey-grey-blue areas are presumably due to a deep melanophagic infiltration. Light brown areas may represent the start of the regression phenomenon, followed by a disarrangement later expressed by reticular blue structures. In lesion zones of MIS corresponding to light brown areas, the disappearance of the rete ridges and the scarcity of intraepidermal melanin may explain the lack of an evident pigment network and the light brown diffuse pigmentation [10]. 

In conclusion, our study provides data about frequency, morphology, extent, and distribution of regression in a large population of MIS of different size. Confirming the findings of our previous paper, we can state that the identification of the reticular pattern of blue regression is a significant discriminator and a reliable predictor of MIS, extremely useful for the diagnosis of early melanoma. 

## Figures and Tables

**Table 1:** Eleven variables of regression and their definitions.

Parameter	Definition
(1) Regression	Presence of grey-blue areas and/or peppering and/or blue-whitish veil and/or white areas

(2) Grey-blue areas	Bluish and/or larger areas of grey-blue pigmentation
(a) Structureless	Grey-blue homogeneous pigmentation
(b) Reticular	Coarse blue-grey net, with thick grey-blue lines and large holes
(c) Globular	Aggregated grey-blue globules

(3) Peppering	Blue, fine pepper-like structures

(4) White areas	White scar-like areas

(5) Blue-whitish veil	Compact, structureless, irregular, and confluent blue-whitish pigmentation. Similar to a superficial veil, palpable over the lesion

(6) Pink areas	Areas of the lesion lighter than the surrounding skin with a pinkish shade

(7) Light brown areas	Light brown structureless irregular areas

(8) Regression of dermoscopic structures	Fading of net, globules, or pigmentation giving rise to light brown areas or small structureless areas within a structured area

**Table 2:** Frequency (%) of regression descriptors in melanoma in situ (MIS).

	MIS (111 = 100%)
Regression present	80.1
Regression of dermoscopic structures	81.9
Light brown areas	81.0
Grey-blue areas	74.7
Pink areas	50.4
Peppering	30.6
White areas	10.8
Blue-whitish veil	1.8

**Table 3:** Frequency (%) of pattern and extent of grey-blue areas in MIS.

Grey-blue areas	MIS showing grey-blue areas (83 = 100%)
Structureless	59.0
Reticular	66.2
Globular	21.6
<10%	19.2
10–50%	54.2
>50%	26.5

**Table 4:** Number of regression parameters in lesions with regression (%).

Number of regression parameters	MIS with regression (89 = 100%)
1	64
2	25.8
≥3	10.1

**Table 5:** Frequency (%) of patterns of regression features according to lesion diameter.

	Lesion diameter	
	≤1 cm (68 = 100%)	>1 cm (43 = 100%)
Regression features	75	88.3
Grey-blue areas (number of lesions ≤1 cm and >1 cm)	70.5	81.3
White/pink areas	36.7	72
